# Supplementary material for: Promoting electrocatalytic CO2 reduction to formate via sulfur-boosting water activation on indium surfaces
Source: Nat Commun. 2019 Feb 21;10:892. doi: 10.1038/s41467-019-08805-x (PMC6385284; doi:10.1038/s41467-019-08805-x)
Supplement: Supplementary file 1 — Supplementary Information [file 41467_2019_8805_MOESM1_ESM.pdf]

Supplementary Information

Promoting electrocatalytic CO_2 reduction to formate via sulfur-boosting water activation on indium surfaces

Ma et al.

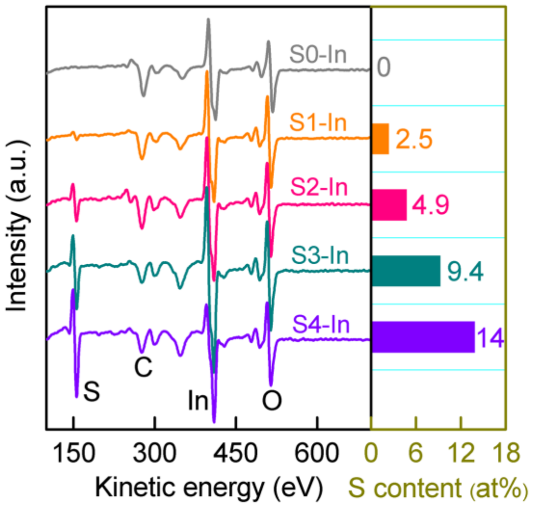

Supplementary Fig. 1 Sulfur contents in S−In catalysts measured by Auger electron spectroscopy.

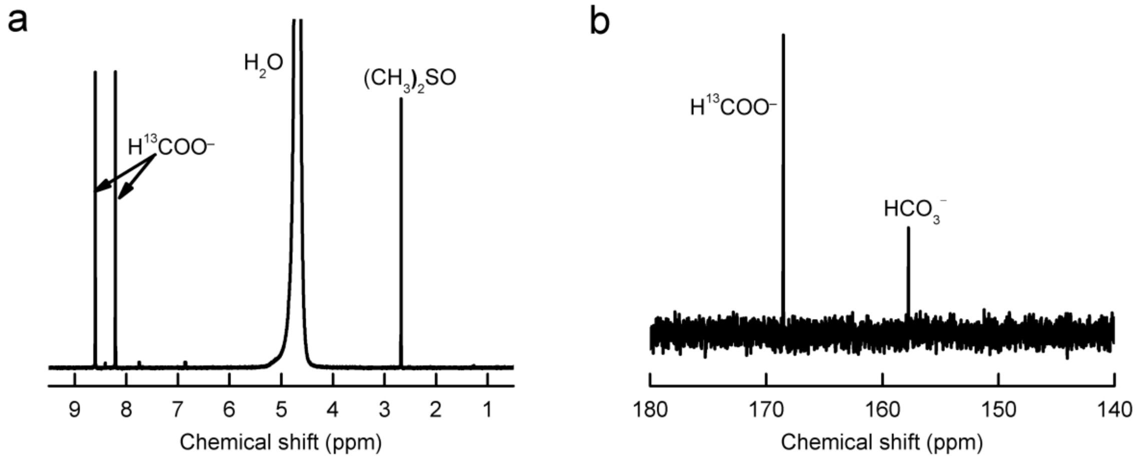

Supplementary Fig. 2 NMR spectra of products for $^{13}CO_2$ labelling experiments over the S2−In catalyst at −0.98 V versus RHE. **a** ^{1}H-NMR spectrum. **b** ^{13}C-NMR spectrum.

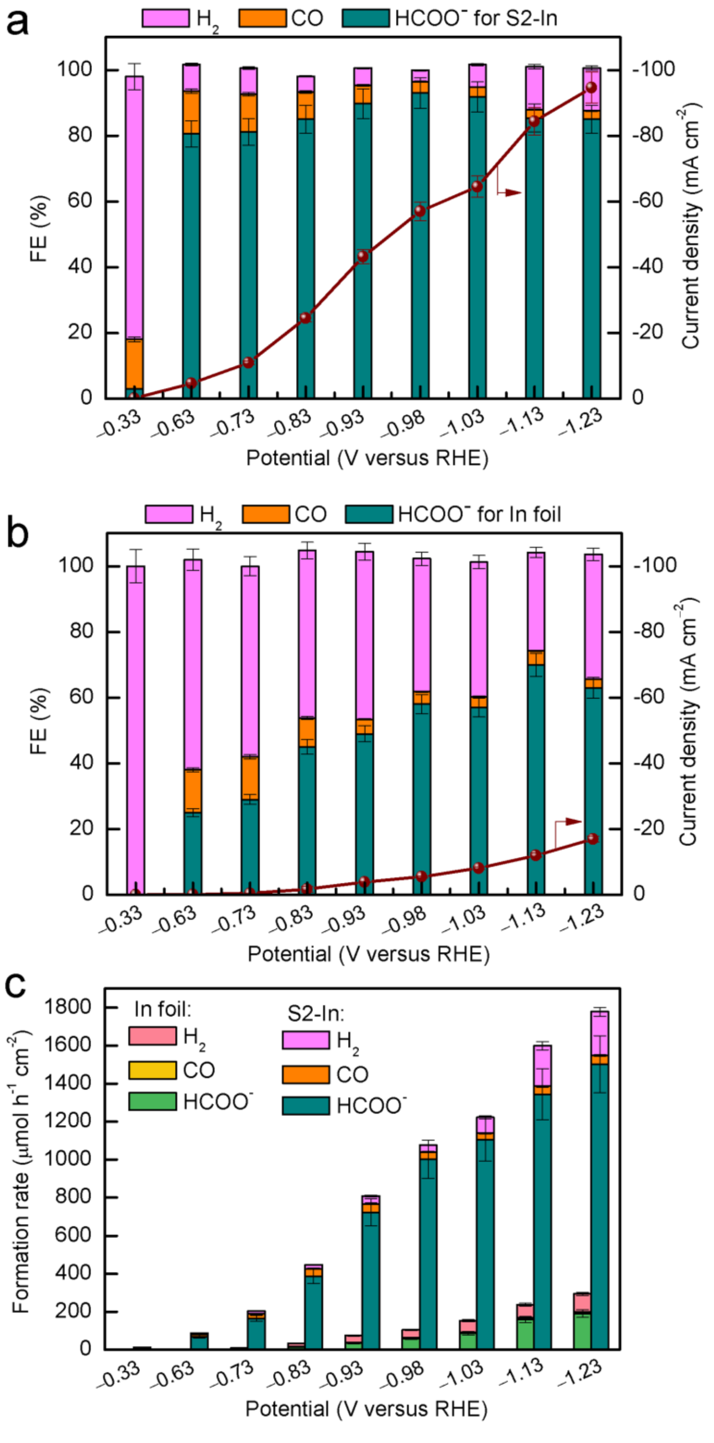

Supplementary Fig. 3 Electrocatalytic CO_2RR performances of the S2−In and In foil catalysts at different applied potentials for 1 h. **a** FEs of H_2, CO, $HCOO^-$ and current density for the S2−In catalyst. **b** FEs of H_2, CO, $HCOO^-$ and current density for In foil. **c** Formation rates of H_2, CO and $HCOO^-$ for both S2−In and In foil catalysts. The experiments in each case were performed at least for three times. The error bar represents the relative deviation.

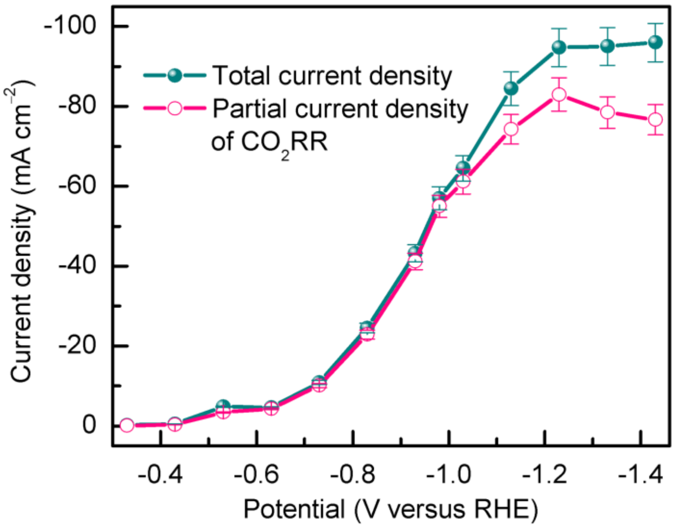

Supplementary Fig. 4 Total current density and partial current density of CO_2RR over the S2−In catalyst at different applied potentials. The partial current density of CO_2RR was calculated by multiplying the total current density by the sum of FEs of formate and CO (CO_2RR products). The experiments in each case were performed at least for three times. The error bar represents the relative deviation.

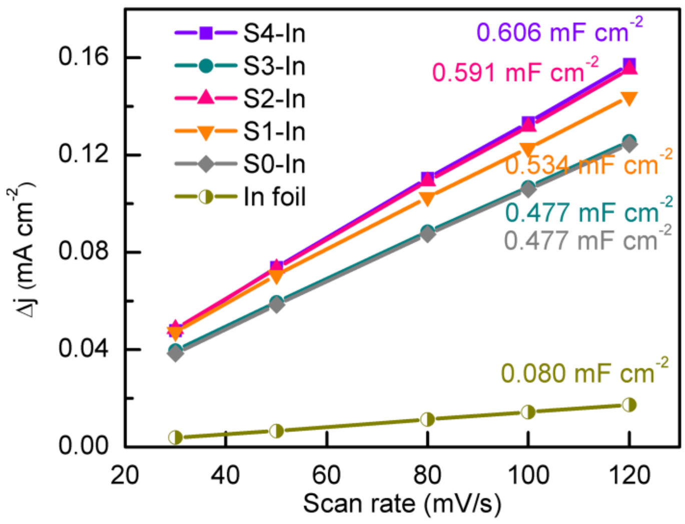

Supplementary Fig. 5 Double-layer capacitance (C_{dl}) of In foil, S0−In, S1−In, S2−In, S3−In and S4−In samples. The C_{dl} was determined by measuring the capacitive current associated with double-layer charging from the scan-rate dependence of cyclic voltammetric stripping. For this, the potential window of cyclic voltammetric stripping was −0.3 to −0.4 V versus SCE (0.5 M $KHCO_3$ solution). The scan rates were 30, 50, 80, 100 and 120 mV s^{-1}. The C_{dl} was estimated by plotting the $\Delta j = (j_a - j_c)$ at −0.35 V (where j_c and j_a are the cathodic and anodic current densities, respectively) versus SCE against the scan rate, in which the slope was twice that of C_{dl}.

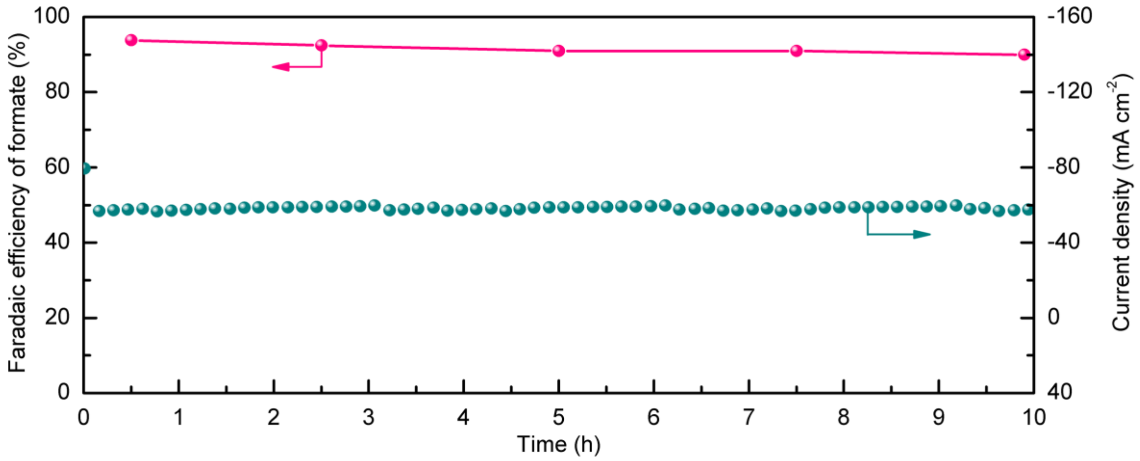

Supplementary Fig. 6 Stability of the S2−In catalyst. The reaction was conducted at −0.98 V (versus RHE) for 10 h in 0.5 M $KHCO_3$ electrolyte in the presence of 0.02 mM of Na_2S.

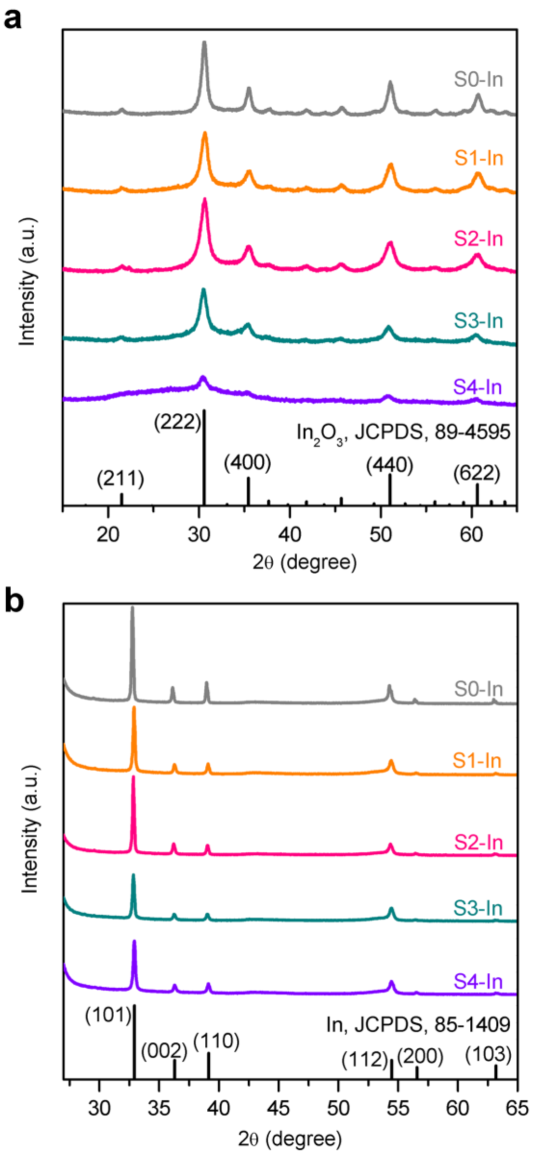

Supplementary Fig. 7 XRD patterns. **a** sulfur-containing In_2O_3 precursors. **b** The S−In catalysts.

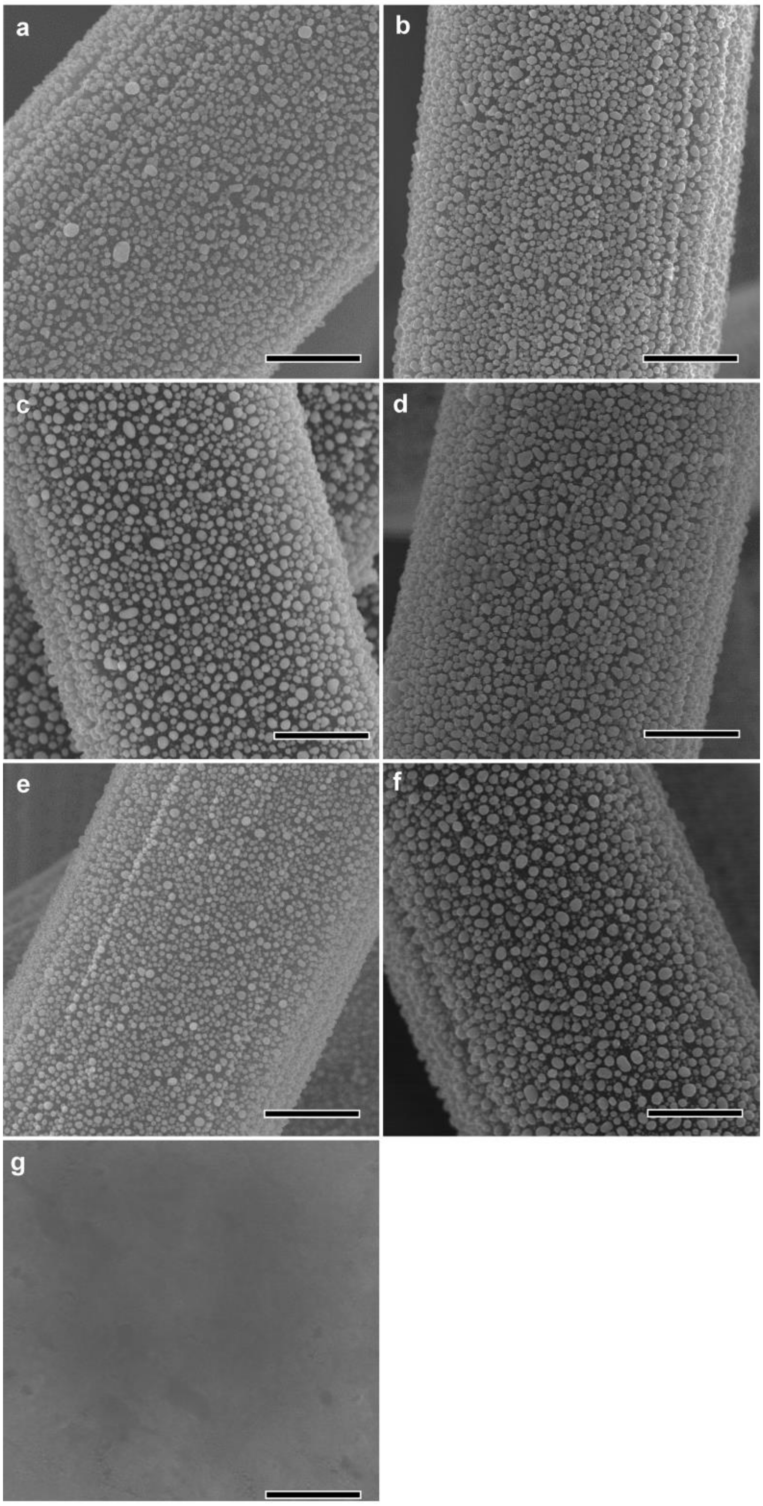

Supplementary Fig. 8 SEM images of the S−In catalysts. **a** S0−In; **b** S1−In; **c** S2−In; **d** S3−In; **e** S4−In; **f** S2−In after reaction at −0.98 V (versus RHE) for 1 h; **g** In foil. Scale bar: 2 μm.

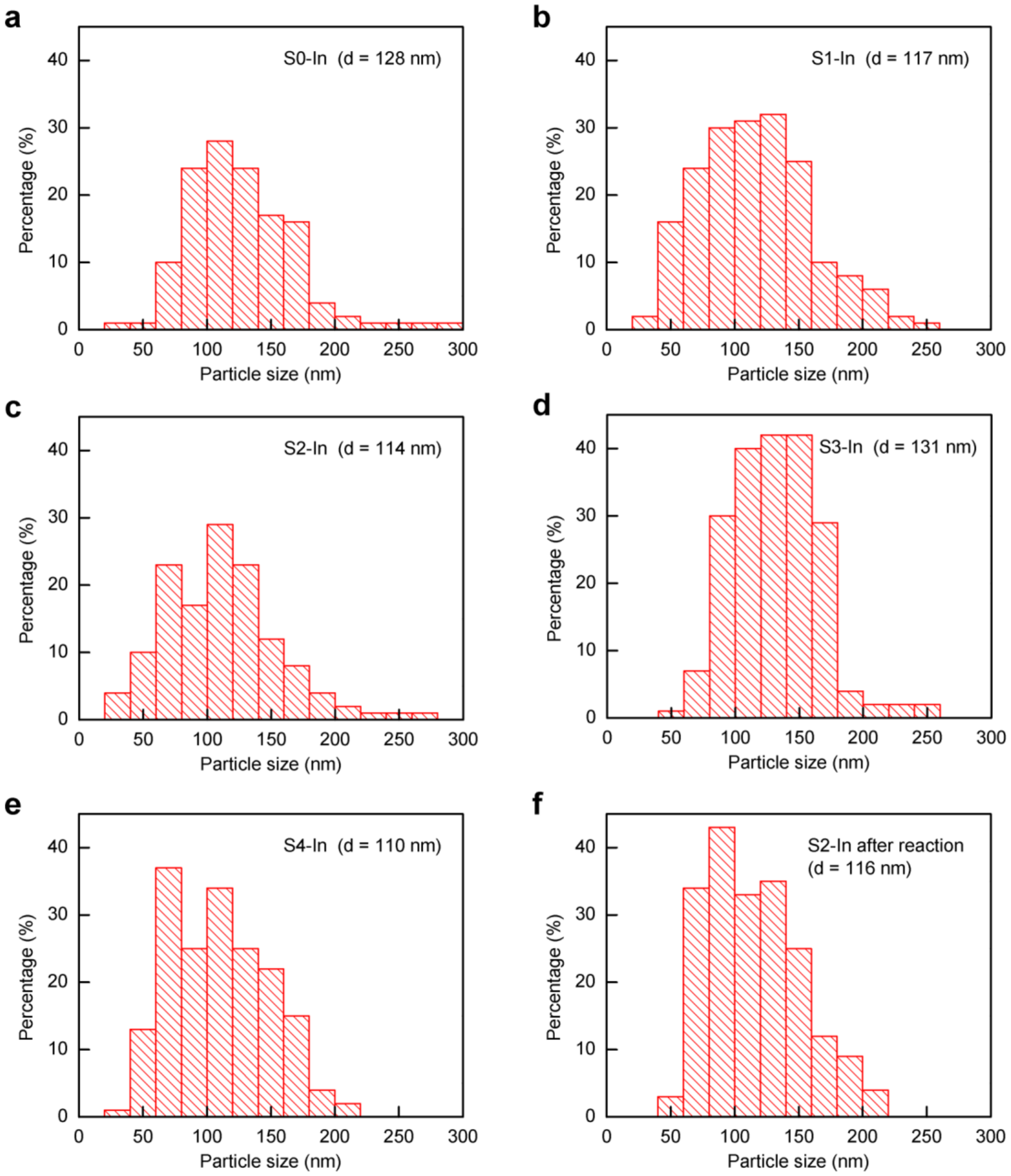

Supplementary Fig. 9 Indium particle size distributions. **a** S0−In; **b** S1−In; **c** S2−In; **d** S3−In; **e** S4−In; **f** S2−In after reaction at −0.98 V (versus RHE) for 1 h.

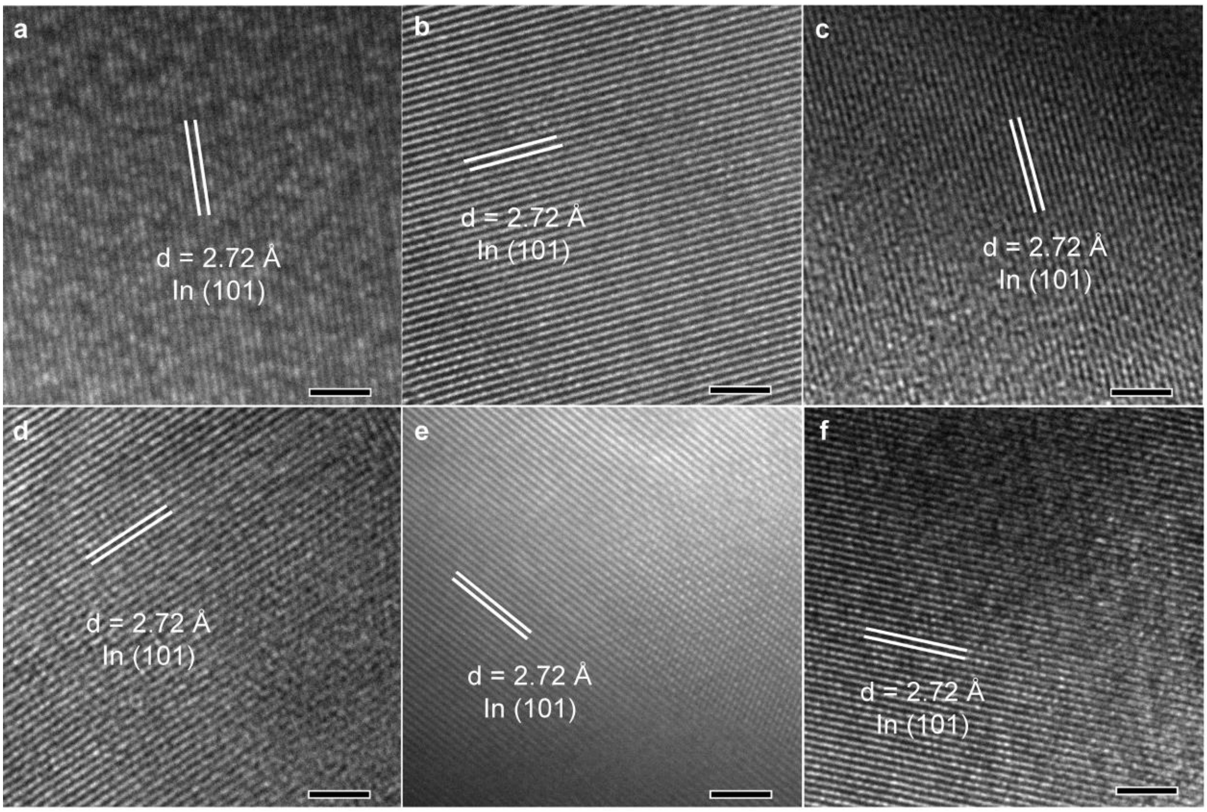

Supplementary Fig. 10 HRTEM images of the S−In catalysts. **a** S0−In; **b** S1−In; **c** S2−In; **d** S3−In; **e** S4−In; **f** S2−In after reaction at −0.98 V (versus RHE) for 1 h. Scale bar: 2 nm.

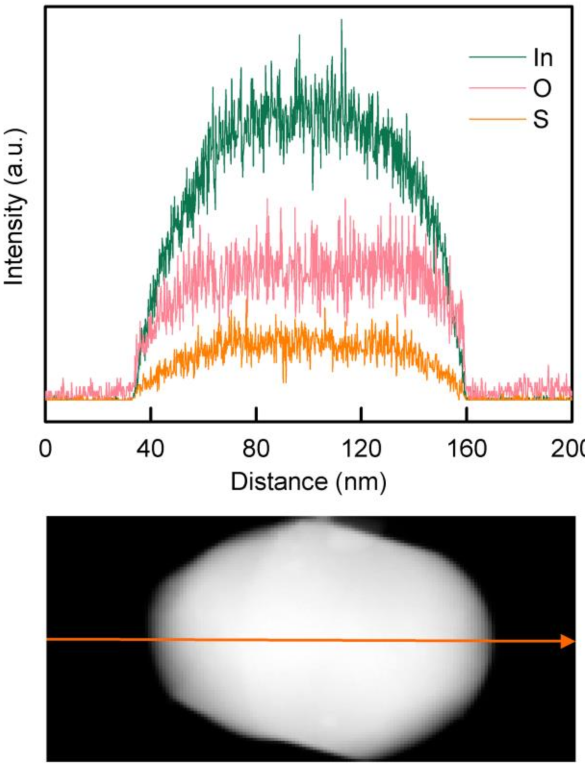

Supplementary Fig. 11 line-scan EDS for the S2−In catalyst.

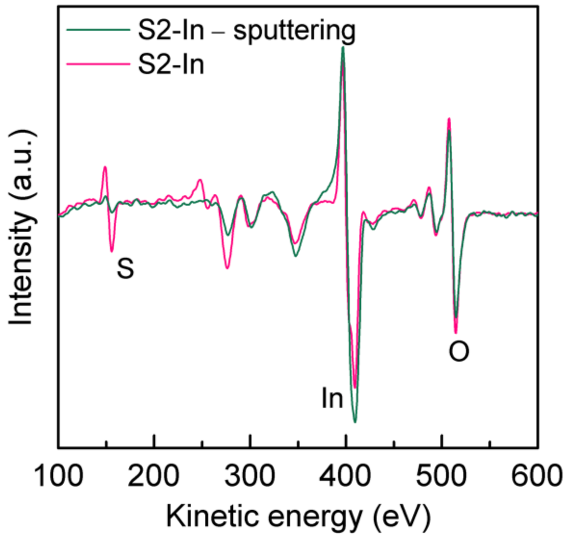

Supplementary Fig. 12 Auger electron spectra before and after sputtering with Ar ions for the S2−In catalyst.

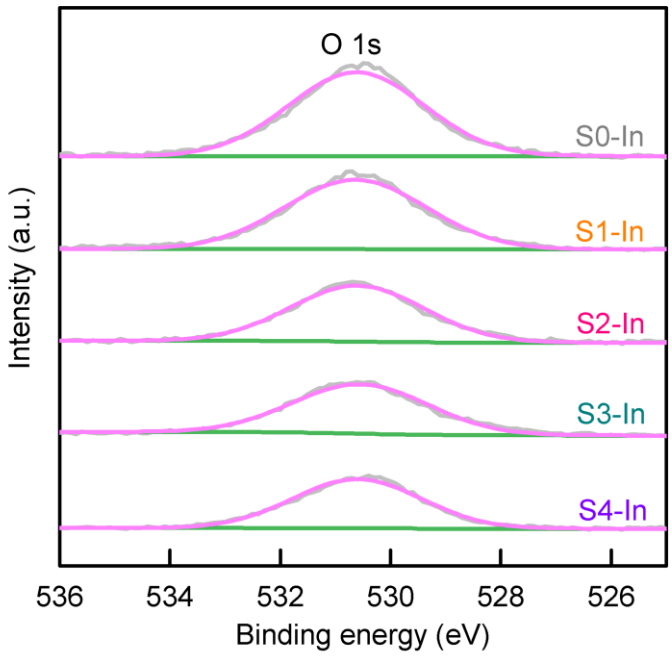

Supplementary Fig. 13 XPS O1s spectra for the S−In catalysts.

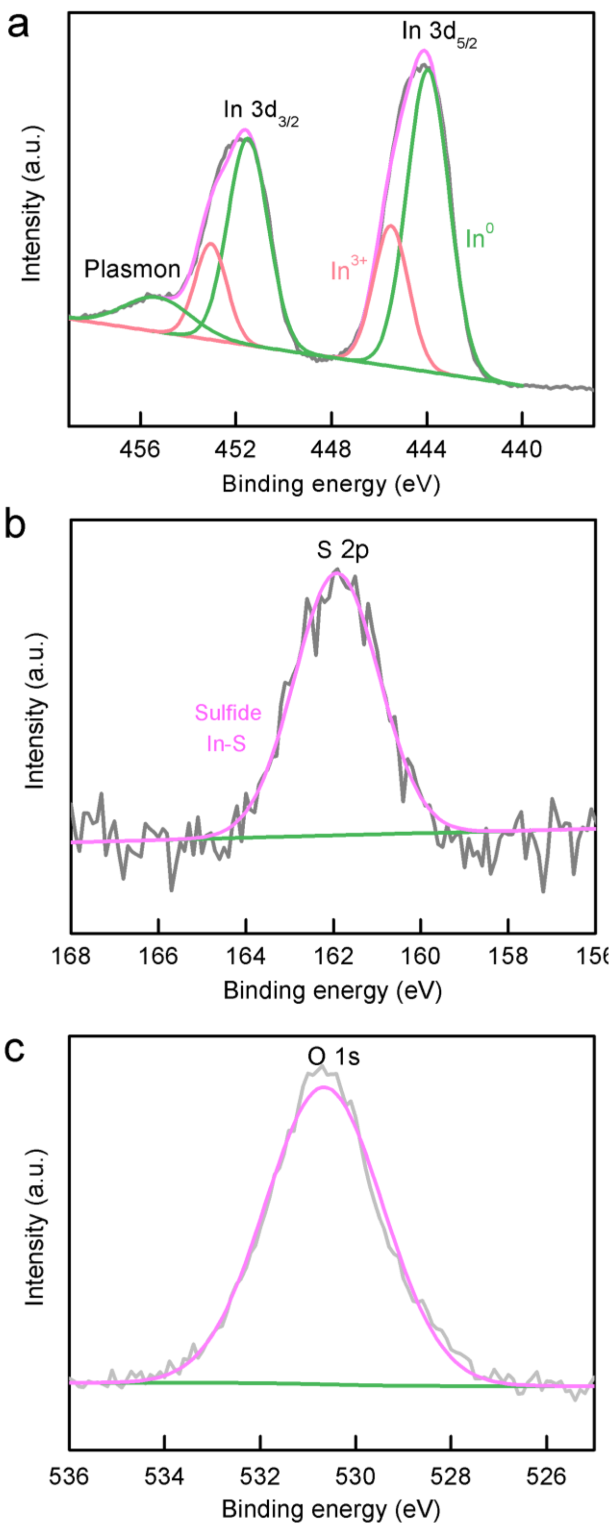

Supplementary Fig. 14 XPS spectra for the S2−In catalyst after electrocatalytic reaction at −0.98 V (versus RHE) for 1 h. **a** In 3d spectrum. **b** S 2p spectrum. **c** O 1s spectrum.

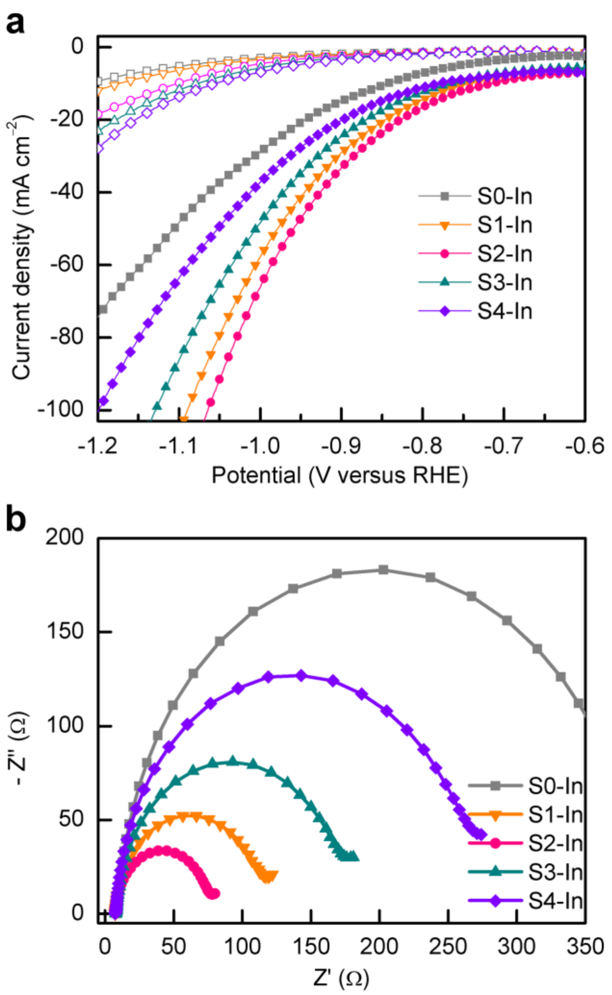

Supplementary Fig. 15 Electrochemical characterization. **a** Linear sweep voltammetric (LSV) curves of S−In catalysts in CO_2-saturated (solid) and N_2-saturated (open) 0.5 M $KHCO_3$ aqueous solution at a scan rate of 50 mV s^{-1}. **b** Electrochemical impedance spectra (EIS) of S−In catalysts. The Nyquist plots were measured with frequencies ranging from 100 kHz to 0.1 Hz at a potential of −0.6 V (versus RHE), and the amplitude of the applied voltage was 5 mV. The impedance data were fitted to a simplified Warburg circuit to extract the charge-transfer resistance.

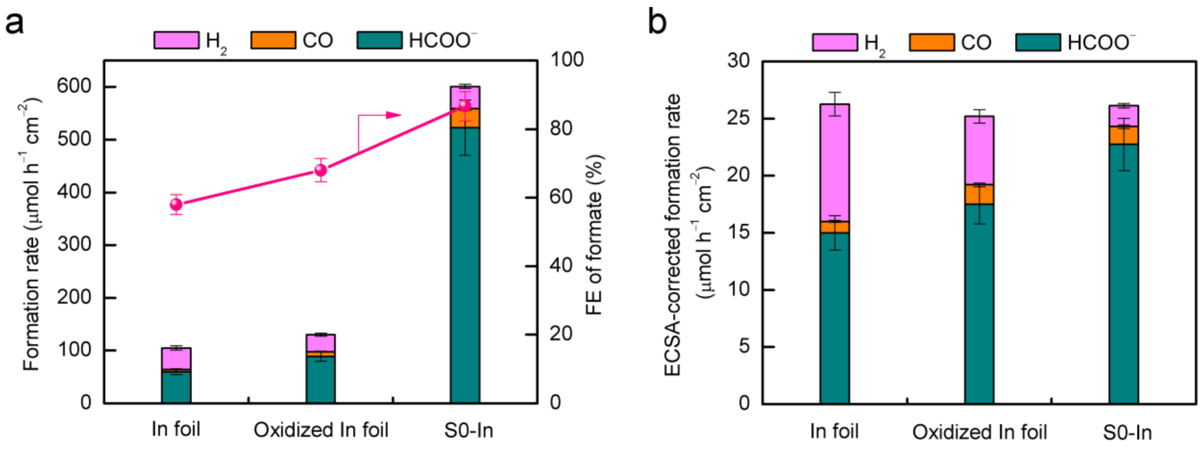

Supplementary Fig. 16 Electrocatalytic performance at −0.98 V (versus RHE) for 1 h**. a** Formation rates of H_2, CO, $HCOO^-$ and FE of formate for In foil, surface-oxidized In foil and the S0−In catalyst. **b** ECSA-corrected formation rates of H_2, CO and $HCOO^-$ for In foil, surface-oxidized In foil and S0−In catalyst. Surface-oxidized In foil was prepared by pretreating In foil in air at 250 ℃ for 3 h. The experiments in each case were performed at least for three times. The error bar represents the relative deviation.

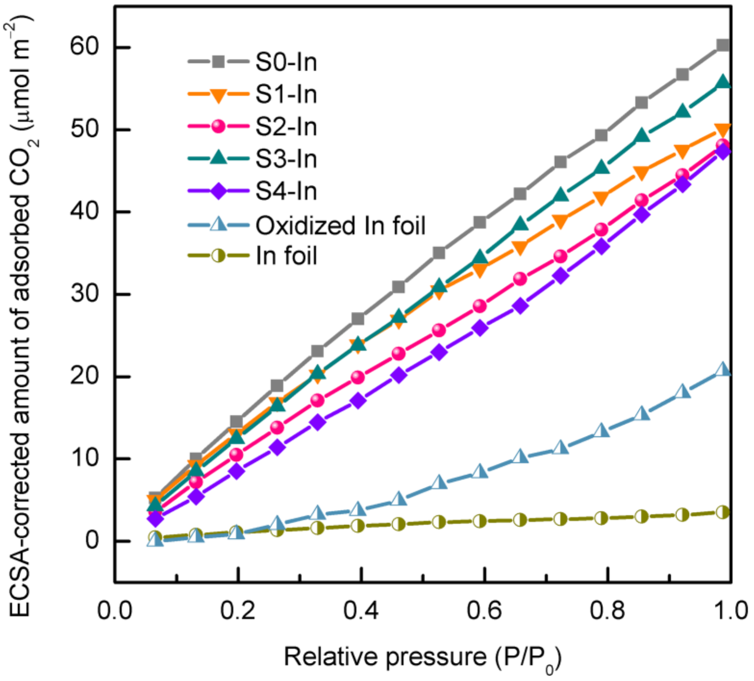

Supplementary Fig. 17 CO_2 adsorption isotherms of In foil, the surface-oxidized In foil and the S−In series of catalysts at 35 ℃.

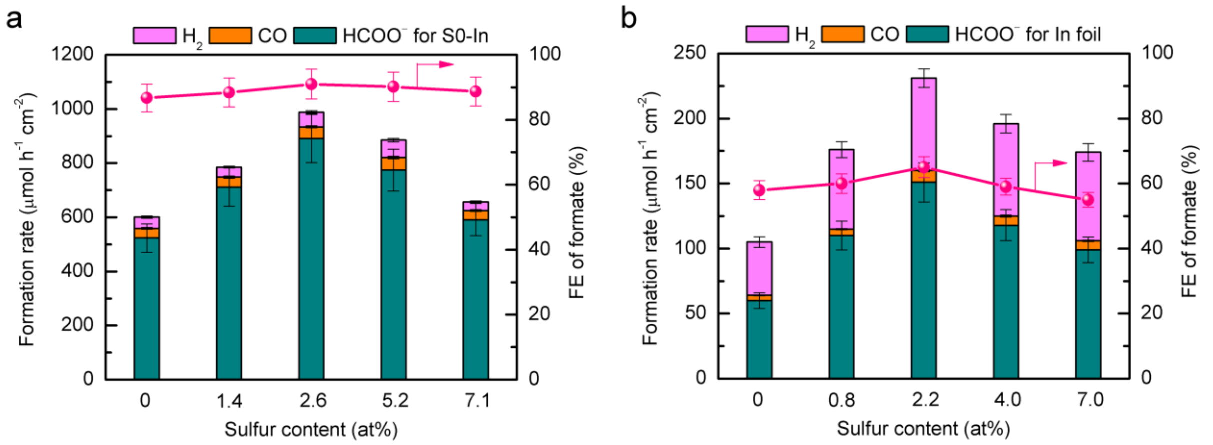

Supplementary Fig. 18 CO_2RR performances for sulfur-modified S0−In and In foil catalysts prepared by impregnation at −0.98 V (versus RHE) for 1 h. **a** Formation rates of H_2, CO, $HCOO^-$ and FE of formate for S-impregnated S0−In catalysts. **b** Formation rates of H_2, CO, $HCOO^-$ and FE of formate for S-impregnated In foil catalysts. The experiments in each case were performed at least for three times. The error bar represents the relative deviation.

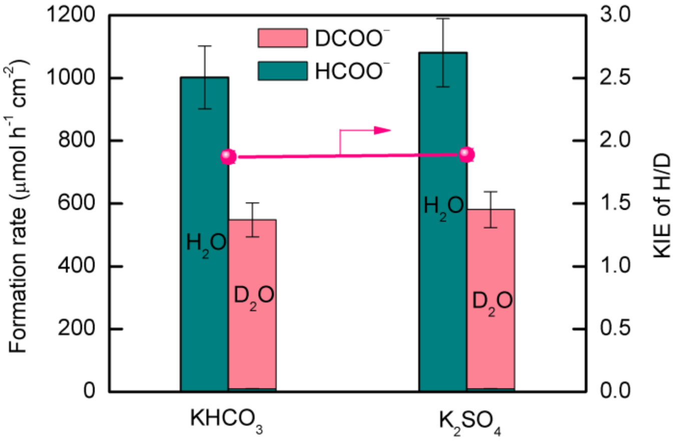

Supplementary Fig. 19 Kinetic isotopic effect (KIE) of H/D over S2−In catalyst at −0.98 V (versus RHE) for 1 h. The experiments in each case were performed at least for three times. The error bar represents the relative deviation.

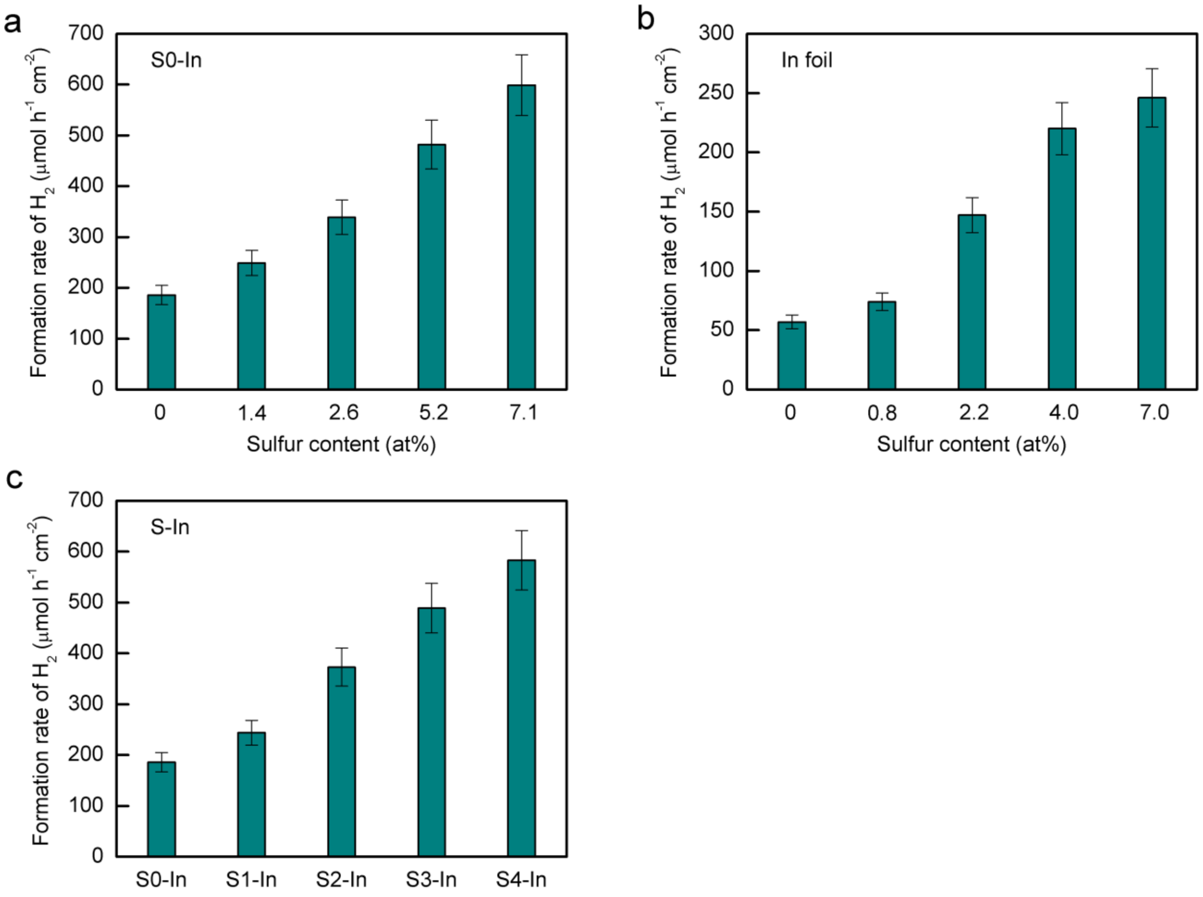

Supplementary Fig. 20 HER performances at −0.98 V (versus RHE) for 1 h. **a** Formation rate of H_2 for S-impregnated S0−In catalysts. **b** Formation rate of H_2 for S-impregnated In foil catalysts. **c** Formation rate of H_2 for S−In series of catalysts. The experiments in each case were performed at least for three times. The error bar represents the relative deviation.

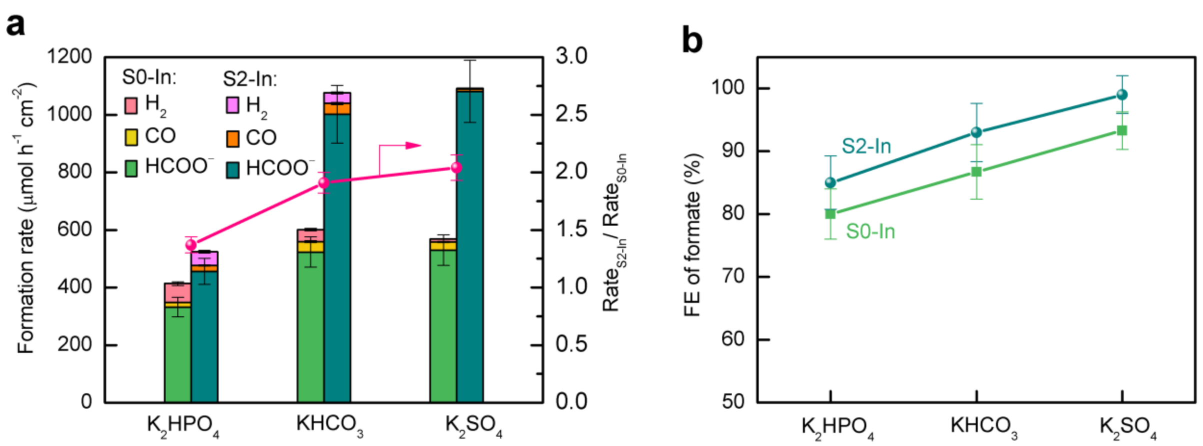

Supplementary Fig. 21 Effect of electrolytes or pH on performances for CO_2RR over the S0−In and S2−In catalysts at −0.98 V (versus RHE) for 1 h. **a** Formation rates of H_2, CO, $HCOO^-$ and the ratio of formate formation rate over the S2−In and S0−In. **b** FE of formate. The experiments in each case were performed at least for three times. The error bar represents the relative deviation.

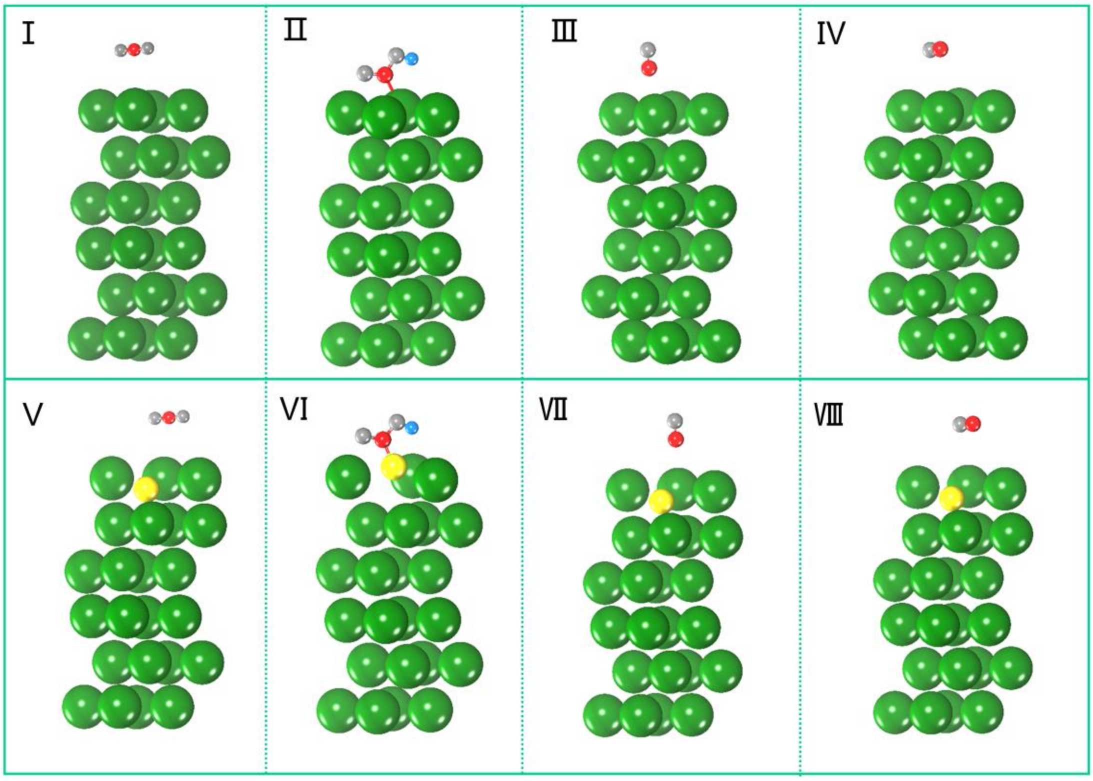

Supplementary Fig. 22 Optimized configurations of I CO_2, II *COOH, III *CO, IV CO on (101) facet of In and V CO_2, VI *COOH, VII *CO, VIII CO on (101) facet S-In. The green, yellow, gray, red and blue balls represent In, S, O, C and H, respectively.

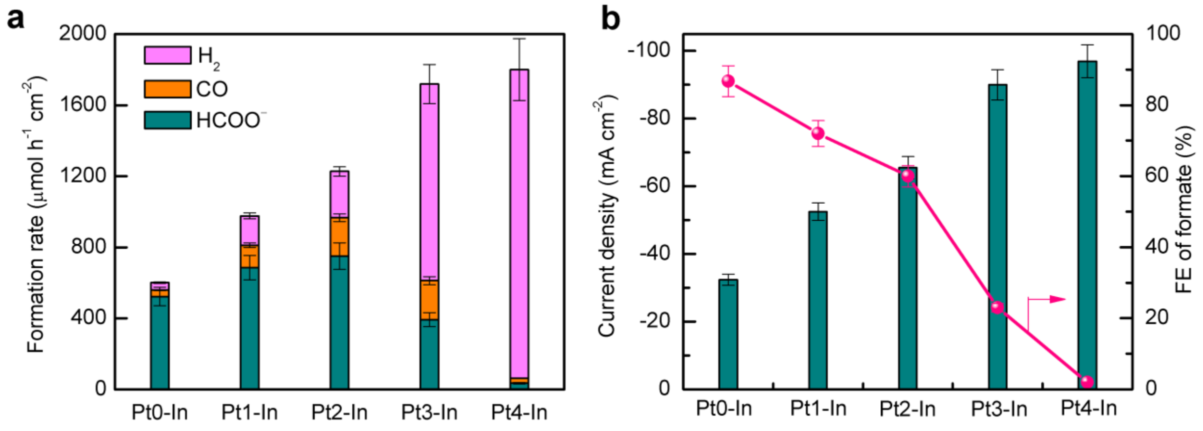

Supplementary Fig. 23 Electrocatalytic CO_2RR performances over Pt-promoted In catalysts at −0.98V (versus RHE) for 1 h. **a** Formation rates of H_2, CO and $HCOO^-$. **b** Average current densities and FE of formate. The experiments in each case were performed at least for three times. The error bar represents the relative deviation.

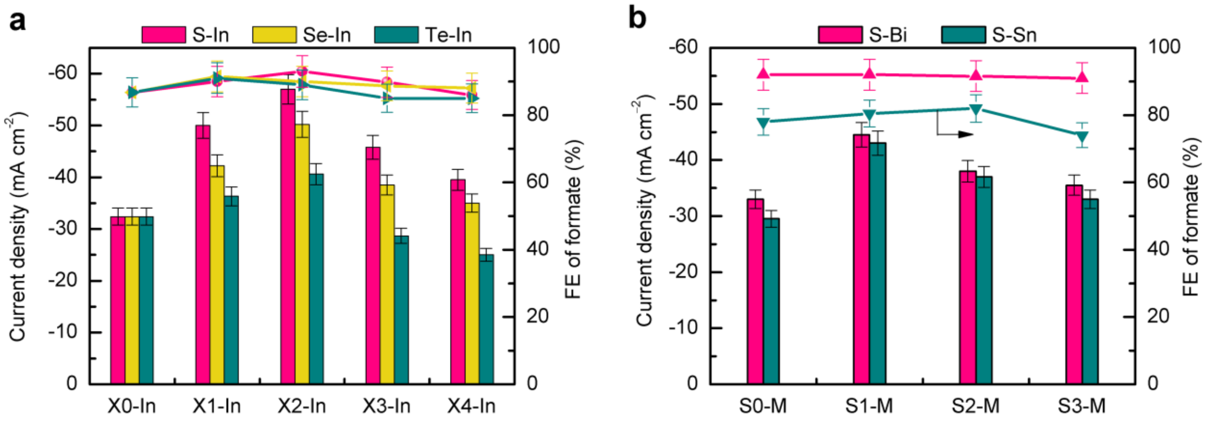

Supplementary Fig. 24 FE of $HCOO^-$ and average current density at −0.98 V (versus RHE) for 1 h. **a** S−In, Se−In and Te−In catalysts. **b** S−Bi and S−Sn catalysts. The experiments in each case were performed at least for three times. The error bar represents the relative deviation.

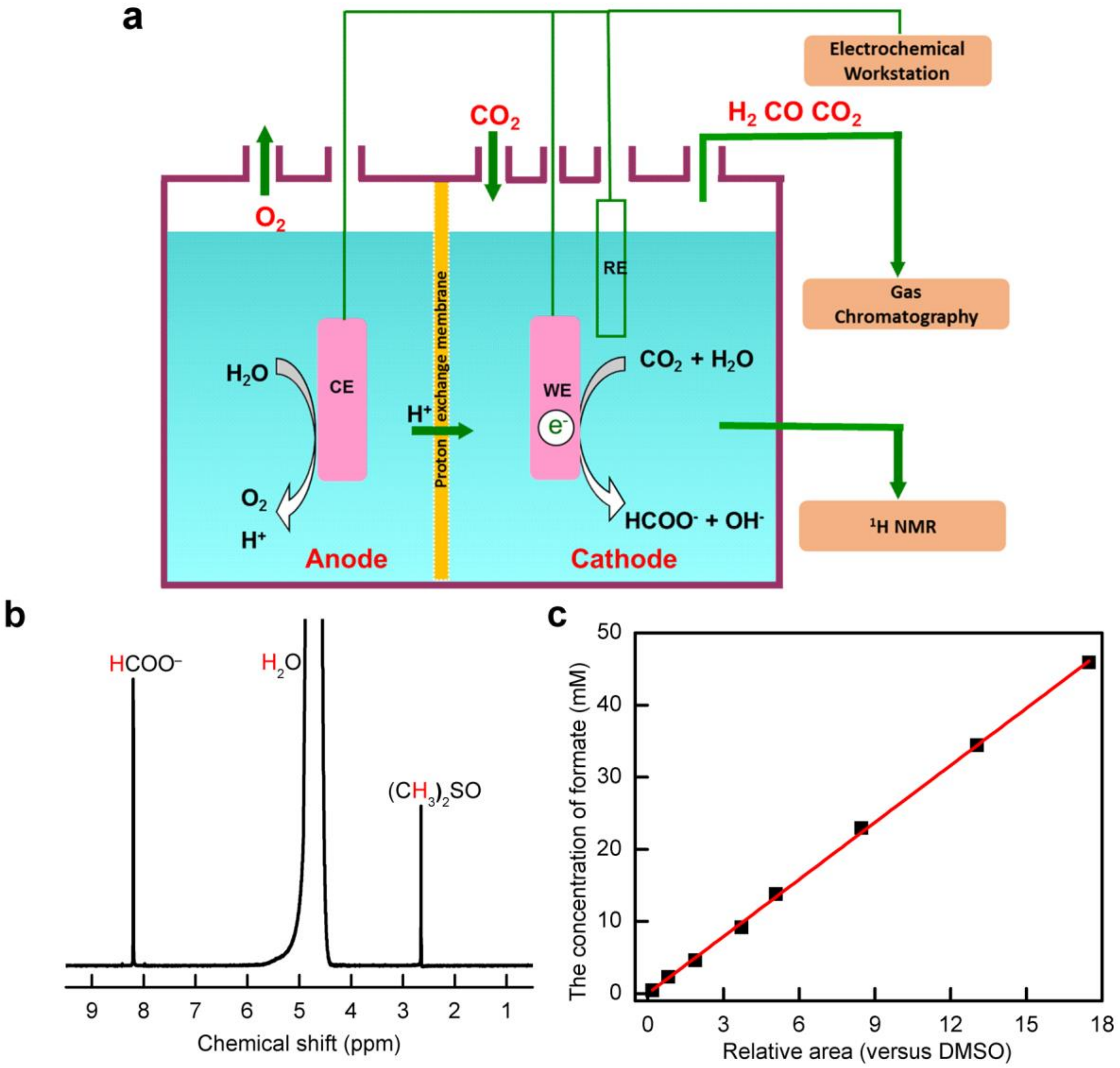

Supplementary Fig. 25 Schematic setup of electrocatalytic apparatus and product measurements. **a** Scheme of the setup for the electrochemical reduction of CO_2. **b** ^{1}H-NMR spectrum for formate determination. **c** Linear relationship between the formate concentration and relative area versus DMSO internal standard.

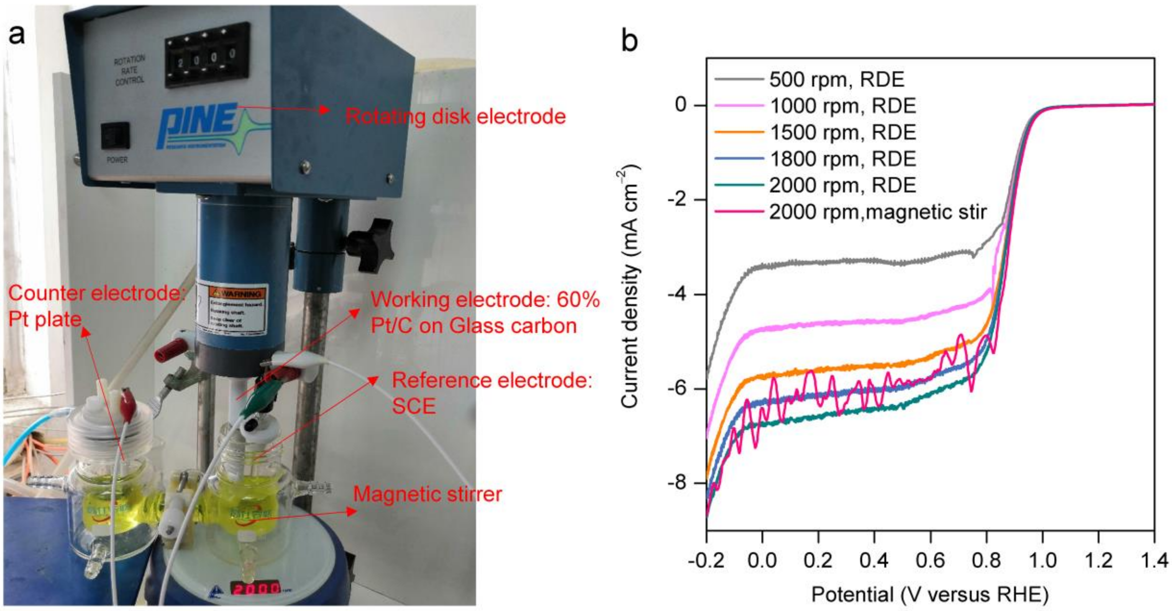

Supplementary Fig. 26 Evaluation of the diffusion layer thickness. **a** Picture of the setup for the determination of the diffusion layer thickness. **b** LSV curves measured at different rotation rates with rotating disk electrode (RDE) or magnetic stirrer with a speed of 2000 rpm.

Supplementary Table 1 Electrocatalytic performances for CO_2 to formate in H-type cell over typical catalysts reported recently

Catalyst	Electrolyte	E_{app} (versus RHE)	j (mA cm^{-2})	$FE_{formate}$ (%)	Formation rate (μmol h^{-1} cm^{-2})	Ref.
Partially oxidized Co	0.1 M Na_2SO_4	−0.25	11	90	178	1
		−0.60	11	~20	~41	
Cu_2O-derived Cu	0.1 M $KHCO_3$	−0.50	2.0	35	13	2
S-modified Cu	0.1 M $KHCO_3$	−0.60	2.5	57	27	3
		−0.80	~15	80	~220	
S-Cu_2O derived Cu	0.1 M $KHCO_3$	−0.65	5.3	64	63	4
		−0.80	10.7	74	146	
ZnO-derived Zn	0.5 M $NaHCO_3$	−1.93	13	87	208	5
In	[Emim]TFA/ D_2O	−1.3	2.0	90	34	6
Anodized In	0.5M K_2SO_4	−0.90	0.3	75	4.2	7
Dendritic In	0.5 M $KHCO_3$	−0.86	5.8	86	93	8
Sn/SnO_x	0.5 M $NaHCO_3$	−0.70	1.8	43	14	9
Porous SnO_2	0.5 M $NaHCO_3$	−0.67	6	35	39	10
		−1.07	50	87	811	
		−1.27	70	~50	653	
SnS_2-derived Sn/rGO	0.5 M $KHCO_3$	−1.05	12	85	186	11
Sn(S)/Au needles	0.1 M $KHCO_3$	−0.75	55	93	957	12
		−1.05	55	94	963	
Sb nanosheets	0.5 M $NaHCO_3$	−0.66	2	50	19	13
		−0.96	8.0	89	132	
Pb	[$BmimPF_6$]/H_2O /AcN	−2.2 (vs. Ag/Ag^+)	41	92	701	14
		−2.5 (vs. Ag/Ag^+)	56	~50	~522	
$Bi_2O_2CO_3$	0.1 M $KHCO_3$	−0.60	1.0	83	15	15
$[Fe_4N(CO)_{12}]^-$	0.1 M Bu_4NPF_6/MeCN	−0.60	3.8	96	68	16
Pd nanoparticles	0.5 M $KHCO_3$	−0.20	22	97	398	17
Pd/C	0.5 M $KHCO_3$	−0.10	4.0	99	74	18
S-In_2O_3 derived In	0.5 M $KHCO_3$	−0.63	5	81	75	This work
		−0.98	57	93	1002	
		−1.23	95	85	1501	
	0.5 M $CsHCO_3$	−0.98	84	93	1449	

Supplementary Table 2 Electrochemical surface area (ECSA) and ECSA-corrected formation rate of formate for different catalysts[a]

Catalyst	ECSA (cm^2)	Formation rate of formate[b] ($\mu mol\ h^{-1}\ cm^{-2}$)	ECSA-corrected formation rate ($\mu mol\ h^{-1}\ cm^{-2}$)
In foil	4	60	15
S0−In	24	523	22
S1−In	27	840	32
S2−In	30	1002	34
S3−In	24	766	32
S4−In	30	637	21

[a] ECSA = $R_f \times S$, in which S stands for the real surface area of the smooth metal electrode, which is equal to the geometric area of carbon fiber electrode (in this work, S = 1 cm^2). The roughness factor R_f was evaluated by the ratio of double-layer capacitance C_{dl} for the working electrode and the corresponding smooth metal electrode (assuming that the average double-layer capacitance of a smooth metal surface is 20 μF cm^{-2}). [b] The reaction was conducted at −0.98 V versus RHE for 1 h in CO_2-saturated 0.5 M $KHCO_3$ electrolyte.

Supplementary Table 3 CO_2RR activity for the S2−In catalyst without and with soaking in 0.1 mM $Zn(NO_3)_2$ solution for 30 s[c]

**Catalyst**	**FE (%)**			**Formation rate (μmol h^{-1} cm^{-2})**			***j***
	H_2	CO	$HCOO^-$	H_2	CO	$HCOO^-$	**(mA cm^{-2})**
S2−In	3.4	3.5	93	37	37	1002	57
S2−In−(Zn^{2+})	3.4	4.2	92.4	26	21	687	40

[c] The electrolysis was conducted at −0.98 V versus RHE for 1 h. Since the reduction potential of ZnS/Zn (as following equation) is more negative than our electrolysis potential, Zn^{2+} is able to block the surface $S^{\delta-}$ by forming stable $Zn^{\delta+}$−$S^{\delta-}$ on the surface.

$$\varphi_{ZnS/Zn} \text{ (versus RHE)} = \varphi^{\Theta}_{ZnS/Zn} \text{ (versus NHE)} + 0.059 \times \text{pH} = -1.47 + 0.059 \times 7.2 = -1.05 \text{ V}$$

Supplementary Table 4 Summary of simulation parameters obtained from DFT calculation

**a** Free energy corrections for gas-phase species (eV)					
Species	E_{DFT}	E_{ZPE}	$\int C_v dT$	TS	G
CO_2	−22.958	0.308	0.091	0.652	−23.211
H_2O	−14.218	0.573	0.104	0.584	−14.125
CO	−14.779	0.136	0.091	0.611	−15.163
HCOOH	−29.879	0.897	0.104	0.988	−29.866
H_2	−6.7665	0.284	0.091	0.403	−6.794
**b** Free energy corrections for surfaces and adsorbates on pure In (eV)					
Species	E_{DFT}	E_{ZPE}	$\int C_v dT$	TS	G
*	−59.941				
*COOH	−85.506	0.599	0.118	0.273	-85.062
HCOO*	-86.730	0.601	0.112	0.244	-86.261
HCOOH*	-89.938	0.892	0.119	0.349	-89.276
*CO	-74.748	0.141	0.118	0.362	-74.851
**c** Free energy corrections for surfaces and adsorbates on sulfur-doped In (eV)					
Species	E_{DFT}	E_{ZPE}	$\int C_v dT$	TS	G
*	−61.798				
*COOH	−88.050	0.669	0.157	0.367	-87.591
HCOO*	-89.057	0.608	0.108	0.230	-88.571
HCOOH*	-92.450	0.902	0.124	0.282	-91.706
*CO	-76.619	0.153	0.132	0.353	-76.687

Supplementary Note 1

Proposed elementary steps involved in the reduction of CO_2 to formate:

$$CO_2 + * \rightarrow CO_2*$$

$$H_2O + e^- + * \rightarrow H* + OH^-$$

$$CO_2* + H* \rightarrow HCOO* + *$$

$$HCOO* + e^- \rightarrow HCOO^- + *$$

Supplementary Methods

Evaluation of mass-transport limited current density

As described in Methods, the current density under mass-transport limitation can be evaluated using the following equation[19]:

$$j_{\text{limit}} = (n \times F \times D \times C) / \delta \qquad (1)$$

(n = 2; F = 96485 C mol^{-1}; D = 2.02 $\times 10^{-9}$ m^2 s^{-1}; C = 34 mol m^{-3} at 1 bar and 25 ℃)

Here, δ is the diffusion layer thickness for CO_2, which can be estimated from rotating disk electrode model with the following Levich equation[19]:

$$\delta = (1.61 \times D^{1/3} \times \upsilon^{1/6}) / \omega^{1/2} \qquad (2)$$

(υ = 1.0 $\times 10^{-6}$ m^2 s^{-1})

Here, ω is the angular frequency of rotation and can be expressed with 2 π $\times$ rotation rate (s^{-1}). Thus, the rotation rate used for the calculation of ω is a key parameter determining the diffusion layer thickness and thus the current density under mass-transport limitation.

To gain the information of diffusion layer thickness under our reaction conditions, we have compared currents between the agitation with magnetic stirrer and the rotation of rotating disk electrode in our H-cell by using linear sweep voltammetry (LSV) measurement (Supplementary Fig. 26a). We used a rotating glassy carbon disc electrode doped with Pt of 3.75 μg cm^{-2} as a working electrode at the same position of cathode for our CO_2RR measurements. The reduction of $K_3Fe(CN)_6$ was chosen to probe the diffusion layer thickness because of its electrochemical reversibility, meaning that the reduction of $K_3Fe(CN)_6$ is facile so that the observed rate is limited only by mass transfer regardless of the applied overpotential[20]. We performed the LSV measurement in 10 mM $K_3Fe(CN)_6$ solution with 0.5 M $KHCO_3$ as an electrolyte at a scan rate of 10 mV s^{-1} from 1.4 to -0.2 V vs. RHE. The current-potential curve was first recorded at the stirring speed of 2000 rpm by magnetic stirrer. Then, the current-potential curves of rotating disk electrode at different rotation rates ranging from 500 to 2000 rpm were recorded to fit the current-potential curve obtained by the magnetic stirrer agitation. The results have been displayed in Supplementary Fig. 26b. The comparison reveals that the current-potential curve for the stirring speed of 2000 rpm is quite close to that for the rotating disk electrode with a rotation rate of 1800 rpm. This result allows us to conclude

that the stirring speed of 2000 rpm in our case is comparable to the rotation rate of 1800 rpm in the rotating disk electrode.

We have calculated the diffusion layer thickness by using the Eq. 1 with a rotation rate of 1800 rpm. The diffusion layer thickness is calculated to be 14.8 μm. Further, the current density under mass-transport limitation for CO_2RR has been calculated to be 90 mA cm^{-2} based on the Eq. 2. This value is in agreement with that reported in literature under similar experimental conditions[21].

Supplementary References

1. Gao, S. et al. Partially oxidized atomic cobalt layers for carbon dioxide electroreduction to liquid fuel. *Nature* **529**, 68-71 (2016).
2. Li, C. W. & Kanan, M. W. CO_2 reduction at low overpotential on Cu electrodes resulting from the reduction of thick Cu_2O films. *J. Am. Chem. Soc.* **134**, 7231-7234 (2012).
3. Shinagawa, T. et al. Sulfur-modified copper catalysts for the electrochemical reduction of carbon dioxide to formate. *ACS Catal.* **8,** 837-844 (2018).
4. Huang, Y. et al. Rational design of sulfur−doped copper catalysts for the selective electroreduction of carbon dioxide to formate. *ChemSusChem* **11**, 320-326 (2018).
5. Zhang, T., Zhong, H., Qiu, Y., Li, X. & Zhang, H. Zn electrode with a layer of nanoparticles for selective electroreduction of CO_2 to formate in aqueous solutions. *J. Mater. Chem. A* **4**, 16670-16676 (2016).
6. Watkins, J. D. & Bocarsly, A. B. Direct reduction of carbon dioxide to formate in high-gas-capacity ionic liquids at post-transition-metal electrodes. *ChemSusChem* **7**, 284-290 (2014).
7. Detweiler, Z. M., White, J. L., Bernasek, S. L. & Bocarsly, A. B. Anodized indium metal electrodes for enhanced carbon dioxide reduction in aqueous electrolyte. *Langmuir* **30**, 7593-7600 (2014).
8. Xia, Z. et al. Highly selective electrochemical conversion of CO_2 to HCOOH on dendritic indium foams. *ChemElectroChem* **4**, 253-259 (2017).
9. Chen, Y. & Kanan, M. W. Tin oxide dependence of the CO_2 reduction efficiency on tin electrodes and enhanced activity for tin/tin oxide thin-film catalysts. *J. Am. Chem. Soc.* **134**, 1986-1989 (2012).
10. Li, F., Chen, L., Knowles, G. P., MacFarlane, D. R. & Zhang, J. Hierarchical mesoporous SnO_2 nanosheets on carbon cloth: a robust and flexible electrocatalyst for CO_2 reduction with high efficiency and selectivity. *Angew. Chem. Int. Ed.* **56,** 505-509 (2017).
11. Li, F. et al. Towards a better Sn: efficient electrocatalytic reduction of CO_2 to formate by Sn/SnS_2 derived from SnS_2 nanosheets. *Nano Energy* **31**, 270-277 (2017).

12. Zheng, X. et al. Sulfur-modulated tin sites enable highly selective electrochemical reduction of CO_2 to formate. *Joule* **1**, 794-805 (2017).
13. Li, F. et al. Unlocking the electrocatalytic activity of antimony for CO_2 reduction by two-dimensional engineering of the bulk material. *Angew. Chem. Int. Ed.* **56**, 14718-14722 (2017).
14. Zhu, Q. et al. Efficient reduction of CO_2 into formic acid on a lead or tin electrode using an ionic liquid catholyte mixture. *Angew. Chem. Int. Ed.* **55**, 9012-9016 (2016).
15. Lv, W. et al. $Bi_2O_2CO_3$ nanosheets as electrocatalysts for selective reduction of CO_2 to formate at low overpotential. *ACS Omega* **2**, 2561-2567 (2017).
16. Taheri, A., Thompson, E. J., Fettinger, J. C. & Berben, L. A. An iron electrocatalyst for selective reduction of CO_2 to formate in water: including thermochemical insights. *ACS Catal.* **5**, 7140-7151 (2015).
17. Klinkova, A. et al. Rational design of efficient palladium catalysts for electroreduction of carbon dioxide to formate. *ACS Catal.* **6**, 8115-8120 (2016).
18. Min, X. & Kanan, M. W. Pd-catalyzed electrohydrogenation of carbon dioxide to formate: high mass activity at low overpotential and identification of the deactivation pathway. *J. Am. Chem. Soc.* **137**, 4701-4708 (2015).
19. Bard, A. J. & Faulkner, L. R. *Electrochemical Methods: Fundamentals and Applications*. (Wiley & Sons, New York, 2001).
20. Clark, E. L. et al. Standards and protocols for data acquisition and reporting for studies of the electrochemical reduction of carbon dioxide. *ACS Catal.* **8**, 6560-6570 (2018).
21. Yan, C. et al. Coordinatively unsaturated nickel–nitrogen sites towards selective and high-rate CO_2 electroreduction. *Energy Environ. Sci.* **11**, 1204-1210 (2018).
